# Risk factors for radiation pneumonitis after rotating gantry intensity-modulated radiation therapy for lung cancer

**DOI:** 10.1038/s41598-021-04601-0

**Published:** 2022-01-12

**Authors:** Saori Tatsuno, Hiroshi Doi, Wataru Okada, Eri Inoue, Kiyoshi Nakamatsu, Masao Tanooka, Masahiro Tanaka, Yasumasa Nishimura

**Affiliations:** 1 Department of Radiation Oncology, Kindai University Faculty of Medicine, 377-2 Ohno-Higashi, Osaka-Sayama, Osaka, 589-8511 Japan; 2 Department of Radiotherapy, Takarazuka City Hospital, 4-5-2 Kohama, Takarazuka, Hyogo Japan

**Keywords:** Cancer therapy, Lung cancer

## Abstract

The risk factors for severe radiation pneumonitis (RP) in patients with lung cancer who undergo rotating gantry intensity-modulated radiation therapy (IMRT) using volumetric modulated arc therapy (VMAT) or helical tomotherapy (HT) are poorly understood. Fifty-two patients who received rotating gantry IMRT for locally advanced lung cancer were included in this retrospective study. In total, 31 and 21 patients received VMAT and HT, respectively. The median follow-up duration was 14 months (range, 5.2–33.6). Twenty (38%) and eight (15%) patients developed grade ≥ 2 and ≥ 3 RP, respectively. In multivariate analysis, lung V5 ≥ 40% was associated with grade ≥ 2 RP (P = 0.02), and past medical history of pneumonectomy and total lung volume ≤ 3260 cc were independently associated with grade ≥ 3 RP (P = 0.02 and P = 0.03, respectively). Rotating gantry IMRT was feasible and safe in patients with lung cancer undergoing definitive radiotherapy. Reducing lung V5 may decrease the risk of symptomatic RP, and care should be taken to avoid severe RP after radiotherapy in patients with a past medical history of pneumonectomy and small total lung volume.

## Introduction

Definitive chemoradiotherapy is a well-established treatment option for unresectable locally advanced non-small cell lung cancer (NSCLC)^1,2^. Recently, the PACIFIC trial demonstrated the efficacy of durvalumab, a human monoclonal antibody against programmed cell death-ligand 1 (PD-L1), in patients with stage III NSCLC as a sequential treatment following standard concurrent chemoradiotherapy^3,4^.

Radiation pneumonitis (RP) is a major complication after thoracic radiotherapy, and grade ≥ 2 RP can occur at the rate of 25–50%^5–8^. Grade ≥ 2 RP after chemoradiotherapy was a significant exclusion criterion in the PACIFIC trial^3,4^. Thus, reducing the risk of RP might maximize the opportunity of receiving consolidation durvalumab, an anti–PD-L1 treatment, based on the PACIFIC trial. However, detailed data on radiotherapy were not collected in the PACIFIC trial.

The RTOG 0617 trial provided significant information for clinical practice, as it was the first large phase III NSCLC study to use intensity-modulated radiotherapy (IMRT) as a treatment modality for locally advanced NSCLC^9^. IMRT was associated with lower rates of grade ≥ 3 pneumonitis in the sub-analysis of the RTOG 0617 trial^10^. In addition, severe pneumonitis was predicted by the lung volume receiving a dose of ≥ 20 Gy (V20), but not V5, which can be increased by IMRT and which has been reported to be a potential risk factor for RP^6,10^. Thus, V20 is a well-established risk factor for RP with high reproducibility^6–8,10^. Further, IMRT can improve target coverage and reduce the volume of normal lungs irradiated with intermediate doses such as V20^11^. Therefore, IMRT is a current standard technique in the definitive radiotherapy for advanced NSCLC.

Several dosimetric parameters for the lungs and risk factors for RP have been reported in patients receiving three-dimensional conformal radiation therapy and IMRT^5,6,8,12^. Volumetric modulated arc therapy (VMAT) and helical tomotherapy (HT) are modern forms of IMRT that are used in clinical practice. Both techniques deliver a radiation beam via a linear accelerator that rotates around the patient and provides low-dose exposure to large volumes of the normal lung. Compared with conventional IMRT using fixed multi-fields, rotating gantry IMRT offers a better dose convergence, fewer monitor units, and a shorter irradiation time^13^. By contrast, a large low-dose irradiated area such as V5 in the lung, which is often caused by IMRT, may be associated with symptomatic RP^6,11^. However, the risk factors for RP in IMRT using VMAT/HT are poorly understood. The purpose of this study was to assess the risk factors for RP in patients receiving definitive radiotherapy using rotational IMRT for lung cancer.

## Methods

This retrospective study was approved by the Ethics Committee Kindai University Faculty of Medicine and Research Ethics Committee, Takarazuka City Hospital (approval nos. R02-043 and 20200601, respectively). Written informed consent for radiotherapy was obtained from all individual participants prior to radiotherapy. Informed consent for this study was obtained in the form of opt-out. This study was conducted in accordance with the Declaration of Helsinki.

Fifty-two patients who received definitive radiotherapy using rotating gantry IMRT for lung cancer between April 2018 and December 2020 were included in this retrospective study. Patients with distant metastasis or a follow-up duration of < 6 months without grade 5 RP were excluded. The treatment devices at Kindai University Hospital were Halcyon® and TrueBeam® (Varian Medical Systems, Inc.), and 31 patients received IMRT using VMAT. The remaining 21 patients received IMRT using HT at Takarazuka City Hospital, which adopted Radixact® (Accuray, Inc.).

In typical cases, the gross tumor volume (GTV) was defined as the primary tumor and lymph node metastasis on computed tomography (CT) or positron emission tomography/CT. The clinical target volume (CTV) was created by adding a 3–8-mm margin to the GTV. Elective nodes were not included in the CTV. The planning target volume (PTV) was created by adding a 5-mm margin to each CTV. The prescribed dose was 2 Gy per fraction for 30 fractions. The radiation dosage was calculated to deliver at least 95% of the prescribed dose with 95% coverage of the PTV. The dose constraints of the National Comprehensive Cancer Network (NCCN) guideline were used in clinical practice at both institutions^1^. The total lung volume was defined as both lungs minus the GTV in this study. The target values of the dose constraint for the total lung receiving 20 Gy (V20) ≤ 35% and V5 ≤ 65%, mean dose (Dmean) to the total lung ≤ 20 Gy, maximum dose to the spinal cord < 45 Gy, and Dmean to the heart ≤ 20 Gy based on the recommendation of the NCCN guideline^1^. The time to toxicity was defined as the interval from the start of radiotherapy to the occurrence of pneumonitis. Respiratory toxicity data were extracted retrospectively from the clinical chart and evaluated using the Common Terminology Criteria for Adverse Events (version 5.0) by radiation oncologists who were blinded to all dosimetric data^14^. The Brinkman index was also calculated by multiplying the number of cigarettes smoked per day by the number of years smoked, which was extracted retrospectively from the clinical chart. Dosimetric parameters for the total lung and contralateral lung were assessed separately in this study based on a hypothesis that the radiation dose of the contralateral lung might affect the development of symptomatic RP.

### Statistical analysis

Data are expressed as the median with the range in parentheses unless otherwise indicated. The cumulative time to RP was calculated using the Kaplan–Meier method. Differences in probability curves were assessed using the log-rank test. The Cox proportional hazards model was used to evaluate factors that influence RP. The cutoffs of possible predictive factors were decided using receiver operating characteristic curves excluding age of 65 years because of the World Health Organization definition of elderly^15^. Results are reported as hazard ratios with their corresponding 95% confidence intervals (CIs). Variables significant at P < 0.05 according to univariate analysis were analyzed in the multivariate model via Cox regression analysis. The Wilcoxon test was used to compare continuous variables and trends among the groups. All statistical analyses were performed using JMP software version 14.0.0 (SAS Institute, Cary, NC, USA). Statistical significance was indicated by P < 0.05.

## Results

Patient characteristics are presented in Table 1. Thirty-eight patients (73%) received at least one course of maintenance immune checkpoint inhibitor treatment following definitive chemoradiotherapy. The median patient age was 70 (range, 53–85). In total, 5 and 47 patients had clinical stage II and III disease, respectively, according to the tumor-node-metastasis classification system (8th edition) of the International Union Against Cancer^16^. Eleven patients had a history of pneumonectomy, and the median lung volume was 2703 cc (range, 696–3799). The median follow-up time was 14 months (range, 5.2–33.6). Eighteen experienced disease failure, and eight patients died during the follow-up period. The median overall survival and disease-free survival were 27.3 and 18.2 months, respectively (Supplementary Fig. S1). In total, 26, 12, 6, 1, and 1 patient developed grade 1, 2, 3, 4, and 5 RP, respectively.

**Table 1:** Patient characteristics.

	n = 52	(%)
Age, years, median (range)	70 (53–85)	
**Gender**		
Male	38	(73)
Female	14	(27)
**ECOG-PS**		
0	28	(54)
1	22	(42)
2	2	(4)
**Smoker**		
Nonsmoker	8	(15)
Smoker (Brinkman index < 500)	17	(33)
Smoker (Brinkman index ≥ 500)	27	(52)
**History of pneumonectomy**		
Yes	11	(21)
No	41	(79)
Total lung volume, median (range)	3038 cc (1609–5533)	
**Laterality of the primary tumor**		
Left	28	(54)
Right	24	(46)
**Location of the primary tumor**		
Upper lobe	27	(52)
Lower lobe	23	(44)
Unspecified	2	(4)
**Pathological diagnosis**		
Adenocarcinoma	27	(52)
Squamous cell carcinoma	16	(31)
Large cell carcinoma	1	(2)
Small cell carcinoma	2	(4)
Others	6	(12)
**Clinical stage**		
IIA	3	(6)
IIB	2	(4)
IIIA	28	(54)
IIIB	15	(29)
IIIC	4	(8)
**Concurrent chemotherapy**^**a**^		
Yes	49	(94)
No	3	(6)
**Maintenance ICI**		
Yes	40	(77)
No	12	(23)
**IMRT**		
VMAT	31	(60)
HT	21	(40)

*ECOG-PS* Eastern Cooperative Oncology Group performance status, *ICI* immune checkpoint inhibitor, *IMRT* intensity-modulated arc therapy, *VMAT* volumetric modulated arc therapy, *HT* helical tomotherapy.

^a^Five patients concurrently receiving immune checkpoint inhibitors were included.

**Figure 1:**
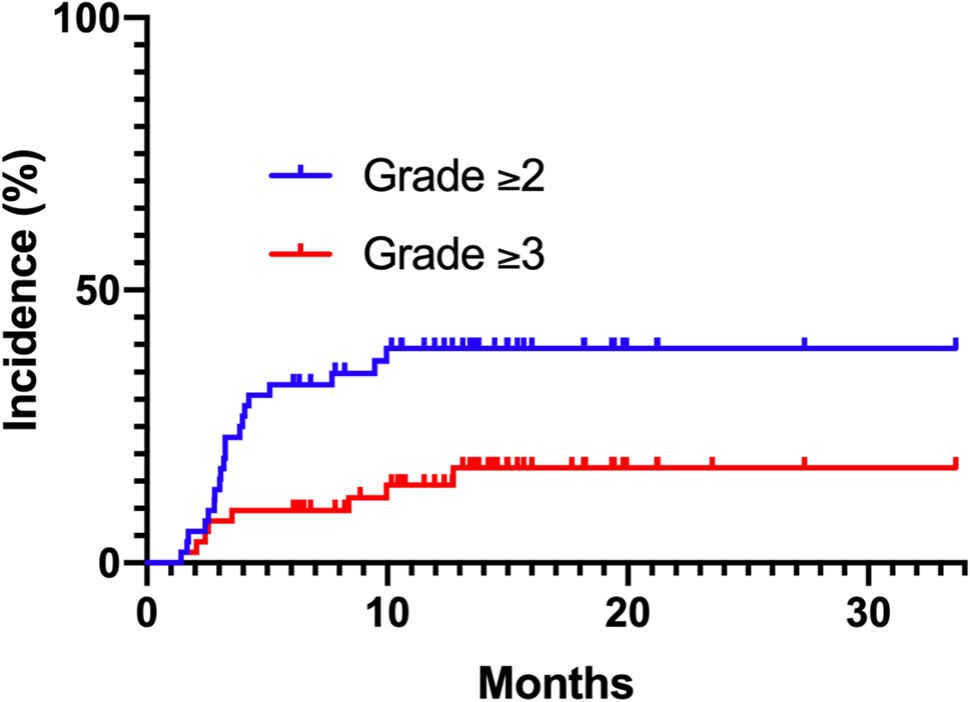
The cumulative incidences of grade ≥ 2 and ≥ 3 radiation pneumonitis (RP). The cumulative incidences of grade ≥ 2 RP were 32.7 and 39.3% at 6 and 12 months, respectively. The cumulative incidences of grade ≥ 3 RP were 9.6 and 14.3% at 6 and 12 months, respectively.

**Figure 2:**
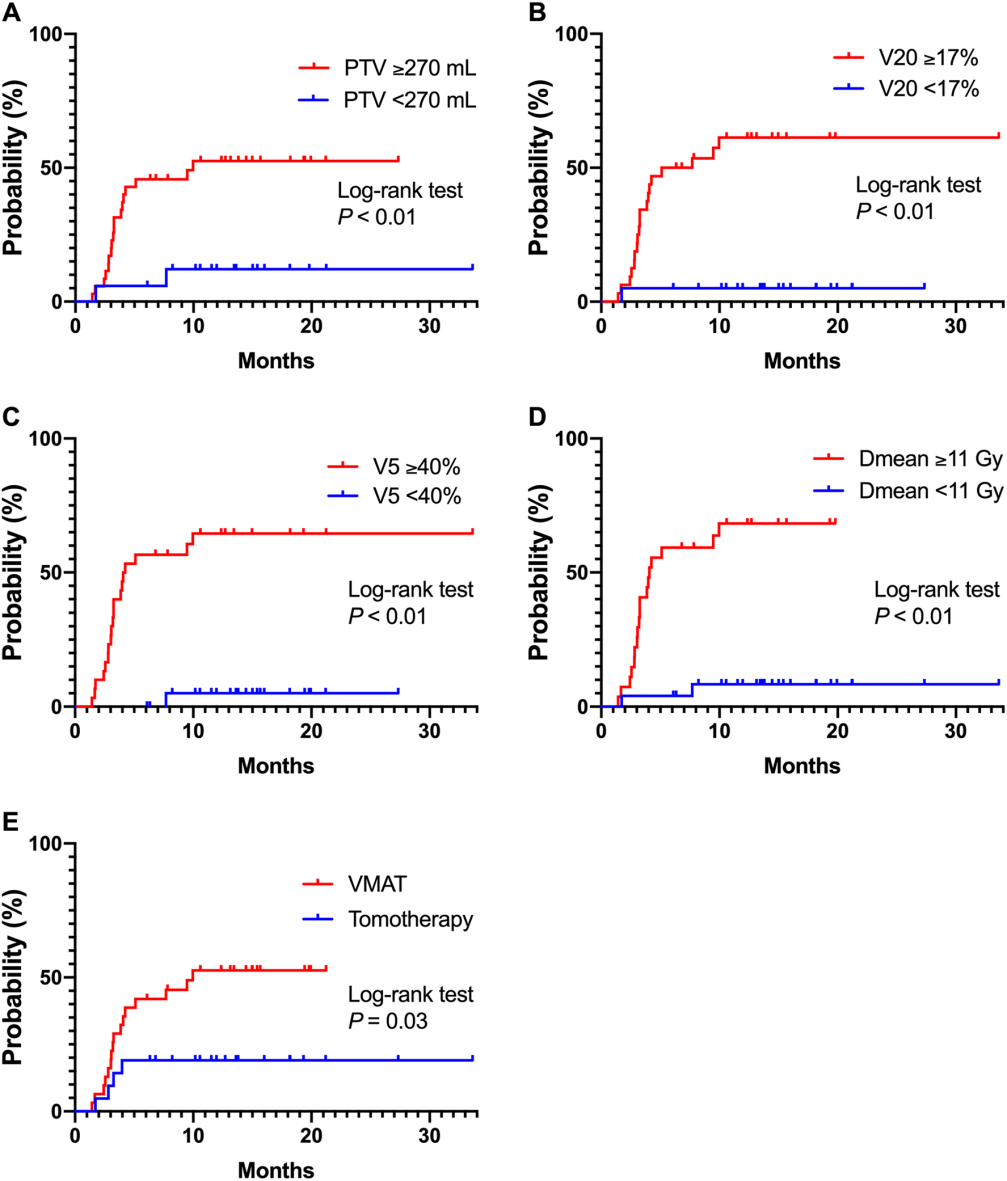
The cumulative incidence of grade ≥ 2 radiation pneumonitis (RP) according to each potential risk factor. The cumulative incidence of grade ≥ 2 RP according to potential risk factor identified as significant in univariate analysis. PTV, planning target volume; V20, lung volume receiving a dose of ≥ 20 Gy; V5, lung volume receiving a dose of ≥ 5 Gy; Dmean, mean dose; VMAT, volumetric modulated arc therapy; HT, helical tomotherapy.

The cumulative incidences of grade ≥ 2 and ≥ 3 RP are indicated in Fig. 1. Univariate analyses revealed that the occurrence of grade ≥ 2 RP was significantly associated with PTV ≥ 270 cc, total lung V20 ≥ 17%, total lung V5 ≥ 40%, total lung Dmean ≥ 11 Gy, and the receipt of VMAT (Table 2). The cumulative incidence of grade ≥ 2 RP according to each risk factor identified as significant in univariate analysis is presented in Fig. 2. In addition, V5 ≥ 40% was associated with grade ≥ 2 RP in multivariate analyses (P = 0.02).

**Table 2:** Univariate and multivariate analyses for occurrence of grade ≥ 2 radiation pneumonitis.

Factors	n = 52	(%)	Univariate analysis		Multivariate analysis	
			Hazard ratio (95% CI)	P-value	Hazard ratio (95% CI)	P-value
**Age (years)**						
< 65	7	14%	1	0.62		
≥ 65	45	87%	0.73 (0.24–3.12)			
**Gender**						
Male	38	73%	1	0.39		
Female	14	27%	1.52 (0.57–3.71)			
**ECOG-PS**						
0	28	54%	1	0.87		
≥ 1	24	46%	0.93 (0.37–2.24)			
**Brinkman index**						
< 500	11	21%	1	0.39		
≥ 500	41	79%	1.66 (0.56–7.11)			
**Laterality of the primary tumor**						
Left	28	54%	1	0.07		
Right	24	46%	2.29 (0.95–5.85)			
**Location of the treated tumor**						
Other lobe	35	67%	1	0.08		
Lower lobe	17	33%	2.24 (0.90–5.43)			
**Previous pneumonectomy**						
No	41	79%	1	0.64		
Yes	11	21%	1.28 (0.42–3.31)			
**Concurrent chemotherapy**						
Yes	49	94%	1	0.08		
No	3	6%	not applicable			
**Maintenance ICI**						
Yes	40	77%	1	0.92		
No	12	23%	0.95 (0.37–2.93)			
**PTV**						
< 270 cc	17	33%	1	< 0.01	1	0.60
≥ 270 cc	35	67%	5.69 (1.64–35.84)		0.49 (0.02–7.54)	
**Total lung volume**						
> 3260 cc	22	42%	1	0.06		
≤ 3260 cc	30	58%	2.47 (0.96–7.61)			
**V20 (total lung)**						
< 17%	20	39%	1	< 0.01	1	0.33
≥ 17%	32	62%	16.85 (3.48–303.28)		4.37 (0.17–115.69)	
**V5 (total lung)**						
< 40%	22	42%	1	< 0.01	1	0.02
≥ 40%	30	58%	21.74 (4.49–391.07)		9.80 (1.48–200.94)	
**Dmean (total lung)**						
< 11 Gy	25	48%	1	< 0.01	1	0.61
≥ 11 Gy	27	52%	12.84 (3.67–81.10)		2.15 (0.11–92.67)	
**IMRT**						
VMAT	31	60%	1	0.02	1	0.25
HT	21	40%	0.31 (0.09–0.86)		0.53 (0.14–1.52)	

*CI* confidence interval, *ECOG-PS* Eastern Cooperative Oncology Group performance status, *ICI* immune checkpoint inhibitor, *PTV* planning target volume, *V20* lung volume receiving a dose of ≥ 20 Gy, *V5* lung volume receiving a dose of ≥ 5 Gy, *Dmean* mean dose, *IMRT* intensity-modulated arc therapy, *VMAT* volumetric modulated arc therapy, *HT* helical tomotherapy.

**Figure 3:**
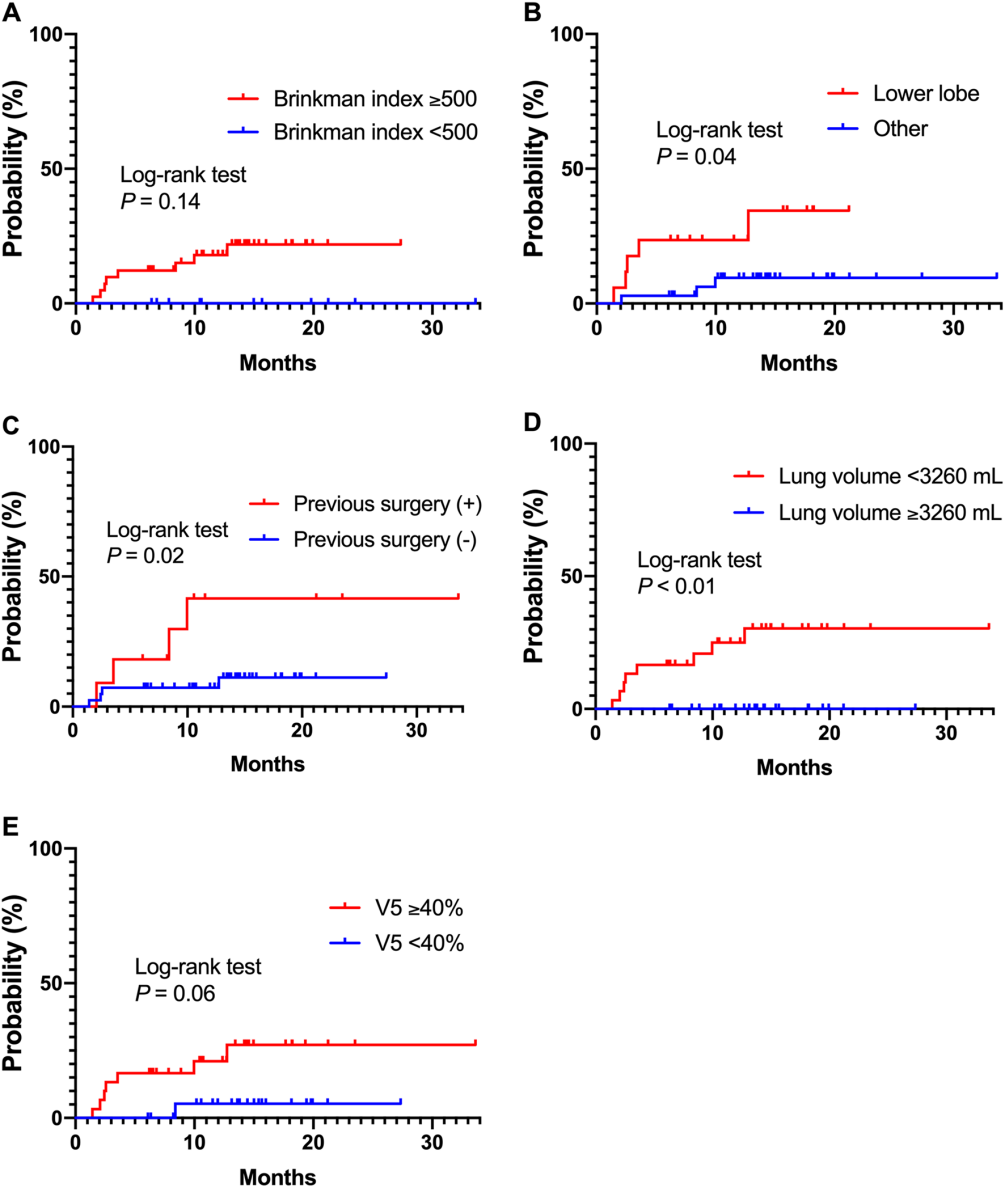
The cumulative incidence of grade ≥ 3 radiation pneumonitis (RP) according to each potential risk factor. The cumulative incidence of grade ≥ 3 RP is presented for each potential risk factor identified as significant in univariate analysis. V5, lung volume receiving a dose of ≥ 5 Gy.

Univariate analyses revealed that the occurrence of grade ≥ 3 RP was significantly associated with Brinkman index ≥ 500, localization of the tumor to the lower lobe, past medical history of pneumonectomy, total lung volume ≤ 3260 cc, and total lung V5 ≥ 40% (Table 3). The cumulative incidence of grade ≥ 3 RP for each risk factor identified as significant in univariate analysis is presented in Fig. 3. In addition, the total lung volume differed between patients with and without a past medical history of pneumonectomy (P = 0.02). In multivariate analysis, a past medical history of pneumonectomy and total lung volume ≤ 3260 cc were significantly associated with the risk of grade ≥ 3 RP (P = 0.02 and P = 0.03, respectively).

**Table 3:** Univariate and multivariate analyses for occurrence of grade ≥ 3 radiation pneumonitis.

Factors	n = 52	(%)	Univariate analysis		Multivariate analysis	
			Hazard ratio (95% CI)	P-value	Hazard ratio (95% CI)	P-value
**Age (years)**						
< 65	7	14%	1	0.94		
≥ 65	45	87%	1.08 (0.19–20.23)			
**Gender**						
Male	38	73%	1	0.30		
Female	14	27%	0.37 (0.02–2.09)			
**ECOG-PS**						
0	28	54%	1	0.85		
≥ 1	24	46%	1.14 (0.27–4.84)			
**Brinkman index**						
< 500	11	21%	1	< 0.05	1	1.00
≥ 500	41	79%	Not applicable		Not applicable	
**Laterality of the primary tumor**						
Left	28	54%	1	0.30		
Right	24	46%	2.15 (0.53–10.47)			
**Location of the treated tumor**						
Other lobe	35	67%	1	< 0.05	1	0.10
Lower lobe	17	33%	4.12 (1.01–20.13)		3.33 (0.78–17.16)	
**Previous pneumonectomy**						
No	41	79%	1	< 0.05	1	0.02
Yes	11	21%	4.43 (1.04–18.94)		6.48 (1.31–35.38)	
**Concurrent chemotherapy**						
Yes	49	94%	1	0.30		
No	3	6%	Not applicable			
**Maintenance ICI**						
Yes	40	77%	1	0.37		
No	12	23%	2.36 (0.42–44.04)			
**PTV**						
< 270 cc	17	33%	1	0.60		
≥ 270 cc	35	67%	1.52 (0.35–10.40)			
**Total lung volume**						
> 3260 cc	22	42%	1	< 0.01	1	0.03
≤ 3260 cc	30	58%	Not applicable		Not applicable	
**V20 (total lung)**						
< 17%	20	39%	1	0.07		
≥ 17%	32	62%	4.99 (0.89–93.45)			
**V5 (total lung)**						
< 40%	22	42%	1	0.04	1	0.06
≥ 40%	30	58%	6.04 (1.07–112.94)		6.16 (0.95–122.66)	
**Dmean (total lung)**						
< 11 Gy	25	48%	1	0.13		
≥ 11 Gy	27	52%	3.19 (0.73–21.84)			
**IMRT**						
VMAT	31	60%	1	0.08		
HT	21	40%	0.21 (0.01–1.16)			

*CI* confidence interval, *ECOG-PS* Eastern Cooperative Oncology Group performance status, *ICI* immune checkpoint inhibitor, *PTV* planning target volume, *V20* lung volume receiving a dose of ≥ 20 Gy, *V5* lung volume receiving a dose of ≥ 5 Gy, *Dmean* mean dose, *IMRT* intensity-modulated arc therapy, *VMAT* volumetric modulated arc therapy, *HT* helical tomotherapy.

Dosimetric parameters for the lungs were compared between patients with and without RP (Table 4). Patients who developed grade ≥ 2 RP received significantly higher V5, V20, and V40 for the total lung than those who did not (all P < 0.01). However, no significant differences were observed in the doses for the total lung between patients with and without grade ≥ 3 RP. In addition, there were no significant differences in the radiation dose for the contralateral lung between patients with and without grade ≥ 2 or grade ≥ 3 RP.

**Table 4:** Comparison of dosimetric values of patients with and without radiation pneumonitis.

Variable	Occurrence of grade ≥ 2 RP			Occurrence of grade ≥ 3 RP		
	Patients with RP	Patients without RP	P-value	Patients with RP	Patients without RP	P-value
**Total lung**						
V5 (%)	52.5 ± 9.4	37.6 ± 15.0	< 0.01	49.8 ± 10.2	42.1 ± 15.5	0.20
V20 (%)	24.1 ± 5.3	16.1 ± 6.8	< 0.01	21.6 ± 5.2	18.6 ± 7.6	0.26
V40 (%)	13.2 ± 4.2	7.9 ± 4.8	< 0.01	11.7 ± 4.7	9.6 ± 5.3	0.24
**Contralateral lung**						
V5 (%)	33.4 ± 14.1	25.9 ± 18.4	0.10	30.5 ± 16.6	28.5 ± 17.4	0.83
V20 (%)	6.1 ± 5.5	5.1 ± 6.4	0.19	4.5 ± 5.3	5.7 ± 6.2	0.82
V40 (%)	1.9 ± 2.4	1.6 ± 2.8	0.27	1.4 ± 2.1	1.8 ± 2.7	0.68

*RP* radiation pneumonitis, *V5* lung volume receiving a dose of ≥ 5 Gy, *V20* lung volume receiving a dose of ≥ 20 Gy, *V40* lung volume receiving a dose of ≥ 40 Gy.

## Discussion

In the present study, risk factors for RP in patients who received definitive radiotherapy using rotational IMRT for lung cancer were investigated. Multivariate analysis revealed that lung V5 ≥ 40% was associated with grade ≥ 2 RP, and a past medical history of pneumonectomy and a small total lung volume were independently associated with grade ≥ 3 RP.

VMAT and HT are modern techniques of rotational IMRT that provide excellent target volume coverage and reduce high doses to normal tissues, but these techniques increase the low-dose areas in the lungs^10^. Current recommended dose constraints for the total lung include V20 < 35% and Dmean < 20 Gy in the NCCN guideline^1^. However, the risk factors for severe RP in patients with lung cancer undergoing VMAT and HT are unclear.

In the present study, the rate of RP after radiotherapy for lung cancer was generally consistent with previous findings^6,17^. Total lung V5 ≥ 40% was an independent predictive factor for grade ≥ 2 RP in multivariate analysis, and it tended to be associated with grade ≥ 3 RP. A large low-dose irradiated area such as V5 in the lung, called a “low-dose bath,” which is often caused by IMRT, has been reported to be associated with symptomatic RP^6,11^. In the present study, total lung V5 was < 65% in most cases. The cutoff of 40% was reproducible in comparison to a previously reported value of 37% for grade ≥ 3 RP^6^. In the RTOG 0617 study, in which IMRT was used in 48.5% of enrolled patients, most patients received V5 > 40%^10^. The reason why no significant association was observed between lung V5 and grade ≥ 3 RP in RTOG 0617 may be that few patients who received V5 < 40% were included in that study. Therefore, the threshold of lung V5 for RP may be lower than the traditional indicator of 65%, and the dose constraint of lung V5 should be lowered to approximately 40% for symptomatic RP.

Grade ≥ 2 RP after chemoradiotherapy was an important exclusion criterion for the administration of maintenance PD-L1 inhibitors following definitive chemoradiotherapy in the PACIFIC study^3,4^. Efforts to reduce the radiation doses to the normal lung may reduce the occurrence of grade ≥ 2 RP and maximize the chance to follow the PACIFIC regimens including consolidation durvalumab following definitive chemoradiotherapy. Conventional constrain for RP is lung V20, and previous reports described that V20 was associated with grade ≥ 3 RP^6,10^. In the present study, the lung dose–volume parameters met the recommended dose constraints for the lungs (V20 and Dmean) in all eligible patients^1^. Rotating gantry IMRT provides a better dose convergence and reduces middle to high dose areas for the lungs, and the occurrence of severe RP can thus be reduced^10,13^. However, the low-dose areas of the lungs can be caused by rotating gantry IMRT^13^. To reduce cases of low-dose bath such as V5, which was a significant predictor of grade ≥ 2 RP in this study, particle therapy could be beneficial in terms of dosimetric parameters for the normal lung ^18,19^. A randomized control study comparing proton therapy and IMRT revealed no significant benefits in terms of the occurrence of RP and locoregional tumor control^20^. However, modern proton techniques such as pencil beam scanning proton therapy with intensity modulation might improve clinical outcomes and provide a dosimetric benefit^19^.

Fatal RP has been reported after thoracic IMRT and HT, and the low-dose area in the contralateral lung has been considered to be associated with the risk of fatal RP^21–23^. However, only a few reports have described the contralateral lung doses during thoracic IMRT. We hypothesized that the contralateral lung should be protected from radiation in treatment planning, and doses were analyzed for both the total lung and contralateral lung in this study. However, no apparent trend was observed regarding the contralateral lung dose between patients who did and did not develop grade ≥ 2 or grade ≥ 3 RP. We consider that limiting the radiation doses for the target lung is more appropriate than protecting the contralateral lung by potentially sacrificing radiation conformity for the target.

Grade ≥ 3 RP is a significant and potentially lethal toxicity of thoracic radiotherapy^9,10^. Previous reports described that V20 was associated with grade ≥ 3 RP^6,10^. In the present study, a previous history of pneumonectomy and a small total lung volume, but not dosimetric parameters, were significantly associated with the risk of grade ≥ 3 RP. In addition, the dosimetric parameters of the total lung or contralateral lung did not differ according to the occurrence of grade ≥ 3 RP. Thus, we consider that the occurrence of grade ≥ 3 RP is related to patient factors. Although patients who experienced nodal recurrence after surgery were included in RTOG 0617, an association between the postoperative status and RP has not been described^9,10^. Concerning the reference value for the total lung volume, previous reports illustrated that the median total lung volume is 3200–3600 cc^6,10^. Therefore, the cutoff of 3260 cc examined in this study may be reproducible. Only a few previous reports documented risk factors for severe RP regarding a history of pneumonectomy and a small total lung volume^24^. We consider that patients with a history of pneumonectomy or a small total lung volume should be considered to have a higher risk of RP.

We acknowledge that our study has several limitations, including its retrospective design and the limited number of eligible patients with heterogeneous characteristics. Because of the limited sample size, grade ≥ 3 RP was not observed in patients with Brinkman index < 500 or total lung volume > 3260 cc in this study. Likewise, CI for lung V5 was large in multivariate analyses for the occurrence of grade ≥ 2 RP because the sample size was limited. Previous pneumonectomy can be a confounding factor if the small total lung volume is associated with grade ≥ 3 RP because there was a significant difference in the total lung volume between patients with and without a past medical history of pneumonectomy. To the best of our knowledge, only a few reports identified the postoperative status and small lung volume as significant risk factors for grade ≥ 3 RP in patients receiving VMAT/ HT. In this study, the dose–volume parameters of the lungs met the recommended dose constraints for lungs (V20 and Dmean) in all eligible patients; thus, the results of the present study appear to reflect current clinical practice^1^. A future study with a larger number of homogenous patients and longer follow-up period is warranted according to our findings.

In conclusion, rotating gantry IMRT was feasible and safe in patients with lung cancer undergoing definitive radiotherapy. As lung V5 ≥ 40% was significantly associated with symptomatic RP, the current dose constraint of lung V5 < 65% might be excessively high for symptomatic RP. In addition, a previous history of pneumonectomy and small lung volume were risk factors for severe RP.

## Supplementary Information

Supplementary Information.

## Data Availability

The data are available from the corresponding authors upon reasonable request.
