# Supplementary material for: Risk factors for radiation pneumonitis after rotating gantry intensity-modulated radiation therapy for lung cancer
Source: Sci Rep. 2022 Jan 12;12:590. doi: 10.1038/s41598-021-04601-0 (PMC8755838; doi:10.1038/s41598-021-04601-0)
Supplement: Supplementary file 1 — Supplementary Information. [file 41598_2021_4601_MOESM1_ESM.pdf]

Supplementary Figure S1.

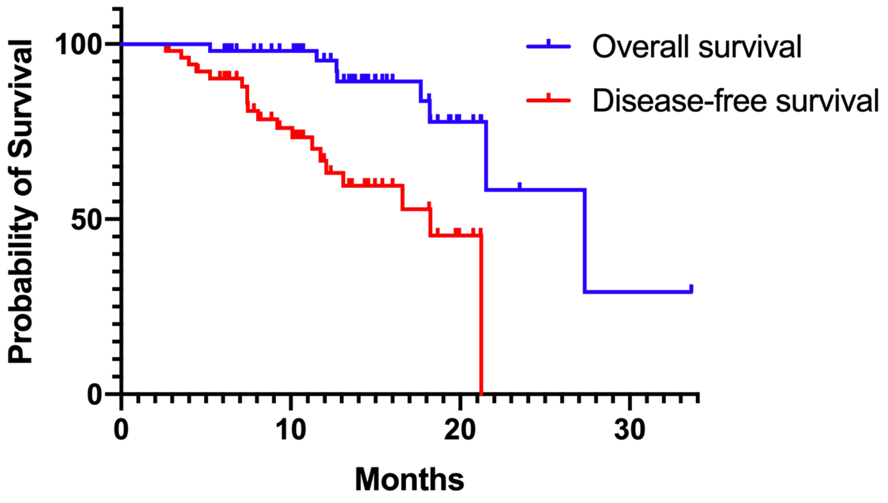

The median overall survival (OS) and disease-free survival (DFS) were 27.3 and 18.2 months, respectively. The 1- and 2-year OS rates were 95.3 and 58.3%, respectively. The 1- and 2-year DFS rate were 63.2 and 0.0%, respectively.
